# Long-Term Changes in Axon Calibers after Injury: Observations on the Mouse Corticospinal Tract

**DOI:** 10.3390/ijms23137391

**Published:** 2022-07-02

**Authors:** Athanasios S. Alexandris, Yiqing Wang, Constantine E. Frangakis, Youngrim Lee, Jiwon Ryu, Zahra Alam, Vassilis E. Koliatsos

**Affiliations:** 1 Department of Pathology, Johns Hopkins School of Medicine, Baltimore, MD 21205, USA; yiqingwa@andrew.cmu.edu (Y.W.); rimmie0714@gmail.com (Y.L.); jiwonr@gmail.com (J.R.); alam.zahra561@gmail.com (Z.A.); 2 Department of Biostatistics, Johns Hopkins School of Public Health, Baltimore, MD 21205, USA; cfranga1@jhu.edu; 3 Department of Neurology, Johns Hopkins University School of Medicine, Baltimore, MD 21205, USA; 4 Department of Psychiatry and Behavioral Sciences, Johns Hopkins University School of Medicine, Baltimore, MD 21205, USA

**Keywords:** axonopathy, traumatic brain injury, white matter microstructure, lognormal distribution

## Abstract

White matter pathology is common across a wide spectrum of neurological diseases. Characterizing this pathology is important for both a mechanistic understanding of neurological diseases as well as for the development of neuroimaging biomarkers. Although axonal calibers can vary by orders of magnitude, they are tightly regulated and related to neuronal function, and changes in axon calibers have been reported in several diseases and their models. In this study, we utilize the impact acceleration model of traumatic brain injury (IA-TBI) to assess early and late changes in the axon diameter distribution (ADD) of the mouse corticospinal tract using Airyscan and electron microscopy. We find that axon calibers follow a lognormal distribution whose parameters significantly change after injury. While IA-TBI leads to 30% loss of corticospinal axons by day 7 with a bias for larger axons, at 21 days after injury we find a significant redistribution of axon frequencies that is driven by a reduction in large-caliber axons in the absence of detectable degeneration. We postulate that changes in ADD features may reflect a functional adaptation of injured neural systems. Moreover, we find that ADD features offer an accurate way to discriminate between injured and non-injured mice. Exploring injury-related ADD signatures by histology or new emerging neuroimaging modalities may offer a more nuanced and comprehensive way to characterize white matter pathology and may also have the potential to generate novel biomarkers of injury.

## 1. Introduction

The unique geometry and energy requirements of axons that make up white matter leave them vulnerable to various insults including hypoxia, oxidative stress and metabolic disturbance, inflammation, and mechanical trauma. As such, white matter pathology is a common occurrence across a wide spectrum of neurological diseases, from traumatic brain injury (TBI) and ischemia to neurodegenerative and neurodevelopmental conditions. White matter pathology has also been the focus for the development of neuroimaging biomarkers that label specific changes in white matter microstructure.

The diameter of axons in the nervous system can vary up to 100-fold (e.g., ∼100 nm–10 μm) and directly relates to conduction velocity, frequency and information transmission rate [1]. The distribution of axon calibers within a single white matter tract is a feature of white matter microstructure intimately related to neuronal function and may be altered in pathological conditions. For example, it has been reported that individuals with autism have a relative deficit in large-caliber axons [2] while reductions of axon calibers in the absence of axonal degeneration has been observed in rat models of chronic alcohol consumption [3]. Moreover, it is recognized that axons are not just the static wire that connects the somatodendritic compartment of neurons to their synaptic terminals but, by actively modulating their calibers, they compute analog and digital signals and optimize the speed of propagation of action potential and hence the temporal transfer of information [4,5,6,7].

While the axon diameter distribution (ADD) of a given white matter tract may be related to function, it is not known to what extend adaptive or pathological processes following injury may also impact on individual axon calibers and ADD. Characterizing early and late changes in ADD may offer novel insights in responses to axonal injury. Here we utilize a mouse model of traumatic brain injury (TBI), i.e., the impact acceleration model (IA-TBI) that is featured by diffuse or traumatic axonal injury (TAI) and leads to the degeneration of several long CNS tracts, to assess early and late changes in ADD post injury. We focus on the injured corticospinal tract (CST) [8,9] and use Airyscan and electron microscopy to determine axon calibers. We find that TAI leads to an early loss of axons of all calibers followed by redistribution of axon diameters in the absence of detectable ongoing degeneration.

## 2. Results

### 2.1. Impact Acceleration TBI Leads to CST Axon Degeneration in the First Week Post Injury

**Figure 1:**
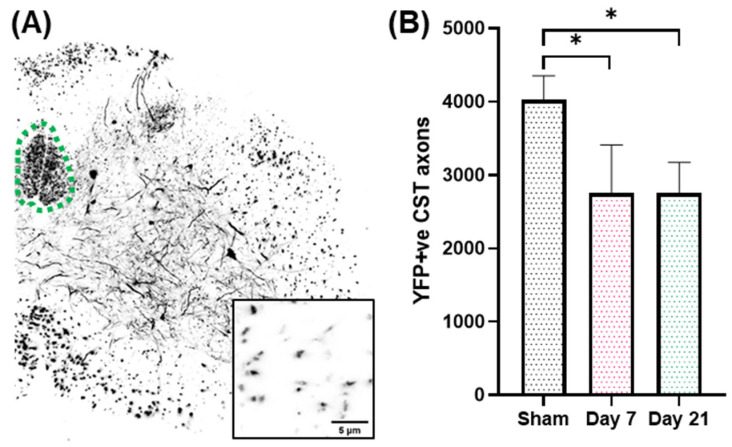
(**A**). Lower cervical spinal cord section showing YFP(+) axons and neurons. The dorsal corticospinal tract is traced with a green dotted line. Inset shows individual CST axons at higher magnification. (**B**). Loss of YFP(+) axons in the CST after single IA-TBI. *, *p* < 0.05.

We have previously shown that IA-TBI results in significant white matter pathology in the spinal cord, including the corticospinal tract (CST). In the acute phase, axonal pathology includes swellings, varicosities, dysmyelination and, in some cases, apparent transection [8,9]. Pathology is primarily encountered in the most caudal pyramids, pyramidal decussation and cervical spinal cord, followed by Wallerian degeneration of the distal axons [8,9]. By examining the CST on semithin and thin sections through the caudal cervical segments at 3 and 7 days post injury, we found that there is no significant increase in pathological burden between 3 and 7 days, indicating that the majority of axon losses occur early after injury [9]. Based on these findings, we further assessed axonal changes in the CST at 7 and 21 days in Thy-YFP-H transgenic mice (*n* = 19) in which CST axons are selectively labeled and can be assessed at single-axon resolution (Figure 1A). In these preparations, IA-TBI results in loss of 30% of axons at both time points (Figure 1B).

### 2.2. Impact Acceleration TBI Leads to Significant ADD Changes

On injured Thy-YFP-H mice, analysis of YFP+ CST axons revealed that the distribution of axon diameters does not follow a normal distribution and has a heavy right-tail skew. Across all cases, the median likelihood ratio for lognormal versus normal distribution was 10^12^ to 1. Therefore, ADDs were fitted with a lognormal model for all experimental groups with R^2^ of 0.94, 0.95 and 0.94 for the sham, day 7, and day 21 groups, respectively.

**Figure 2:**
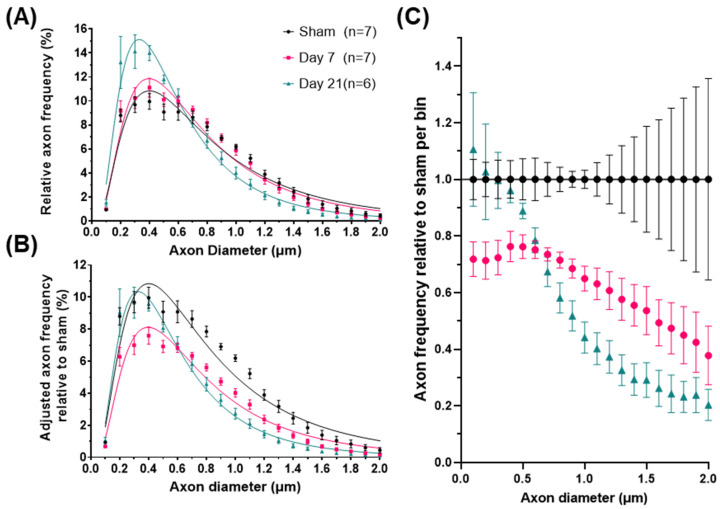
Axonal diameter distribution changes in the corticospinal tract after impact-acceleration traumatic brain injury. (**A**) Relative frequency plots of axon calibers (Feret’s diameter) at 7 or 21 days after injury or sham injury, fitted with lognormal curves. (**B**) Adjusted frequencies relative to the sham injury group (area under the curve represents total axon survival). (**C**) Estimated relative axon frequency per axon diameter bin, compared to sham-injured animals. Error bars represent standard error of the mean for each group.

Comparison of axon diameters between sham and injured subjects revealed significant changes in ADDs primarily due to a progressive decrease in the relative frequency of axons larger than 0.7 μm (Figure 2A). To further describe the changes across groups, we estimated particular shape features of the ADD for each group (Table 1) including the geometric mean (GM) which is equivalent to the median (50th percentile), the geometric standard deviation (GSD) which describes the spread of the distribution and directly relates to its skewness (the degree of asymmetry and tailed-ness of the ADD), as well as the 90th percentile (an index for the large-caliber axons), and the mode (the most frequent axon caliber). All these features indicate that IA-TBI is associated with progressive changes in axons, namely a reduction in overall axon calibers but also in their dispersion and the right-tail of the ADD, i.e., a greater loss in large-caliber axons. For statistical comparisons between groups, we estimated the two core ADD features (GM and GSD) for individual animals and assessed them with one-way ANOVA. There were significant changes across groups for both GM (F_2,17_ = 6.25, *p* = 0.007) and GSD (F_2,17_ = 5.08, *p* = 0.013). While there were no significant differences in the ADD between sham and Day 7 animals (GM: t = 0.98, *p* = 0.4; GSD: t = 1.68, *p* = 0.13), there were significant differences between sham and Day 21 animals (GM: t = 3.39, *p* = 0.003; GSD: t = 2.83, *p* = 0.01), as well as between Day 7 and Day 21 animals for GM (t = 2.64, *p* = 0.019) thought not for GSD (t = 1.88, *p* = 0.087). Assessment of myelinated axon diameters from a separate EM cohort revealed the same pattern (Supplementary Figure S1).

**Figure 3:**
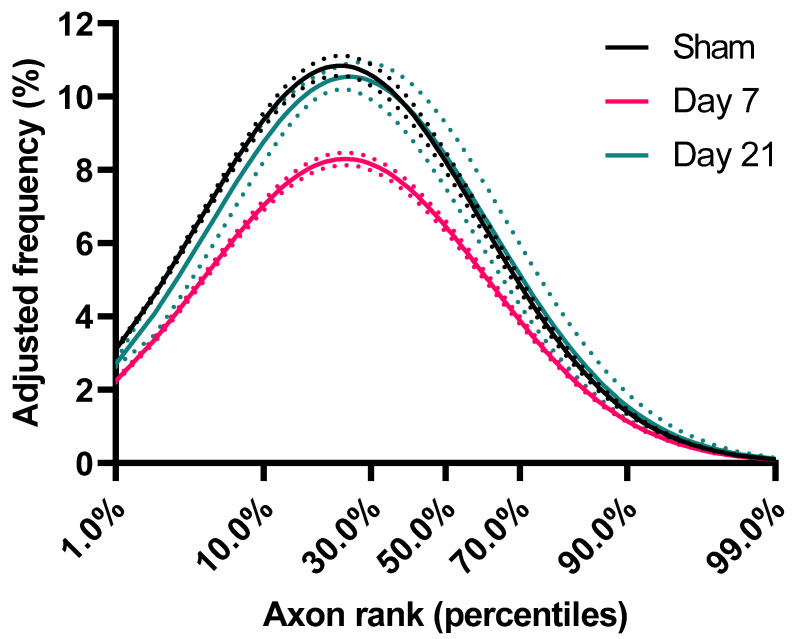
Frequency distribution of axons based on ranking. For each group, axon caliber ranks (percentiles) were calculated and shown against estimates of their corresponding frequencies (adjusted for axon losses). These estimates were imputed from the lognormal curves of the underlying ADD (as per Figure 2B). Dotted lines represent standard error of the mean.

Although the above analysis demonstrates the impact of injury on the shape of the ADD, it does not take into account the loss of axons. Therefore, in order to better understand injury-related changes in populations of axons of different diameters, we calculated an adjusted shape of ADD of each group based on average axon survival (Figure 2B). For example, for the day 7 group, the adjusted frequency plotted for each bin in Figure 2B is the one in Figure 2A multiplied by the proportion of surviving axons in that bin at Day 7. In that group, Figure 2C shows that there is loss of axons of all calibers, although for axons larger than 0.7 μm (representing 44–52% of the initial axon population) axon loss appears proportional to axon diameter (Spearman’s ρ = −0.98, CI_95%_ = −0.99 to −0.95). By day 21 and in the absence of further detectable degeneration (Figure 1B), we observed a significant remodeling of the surviving axonal population. This was indicated by further reductions in the frequencies of axons larger than 0.7 μm and a reciprocal increase in the proportion of small diameter axons, to nearly pre-injury levels (Figure 2C). In order to distinguish the effect of the redistribution in axon frequencies from the overall reduction in diameters, we further ranked axons based on their diameters as percentiles and plotted their corresponding frequencies (Figure 3). We found that, in all groups, axons ranking at the 24–26th percentiles have the highest frequencies, and that early after injury the frequencies of axons up to the 70th percentile are substantially reduced. However, by day 21, the frequencies of axons across ranks are virtually restored to their baseline.

### 2.3. ADD Injury Signatures in Individual Mice

**Figure 4:**
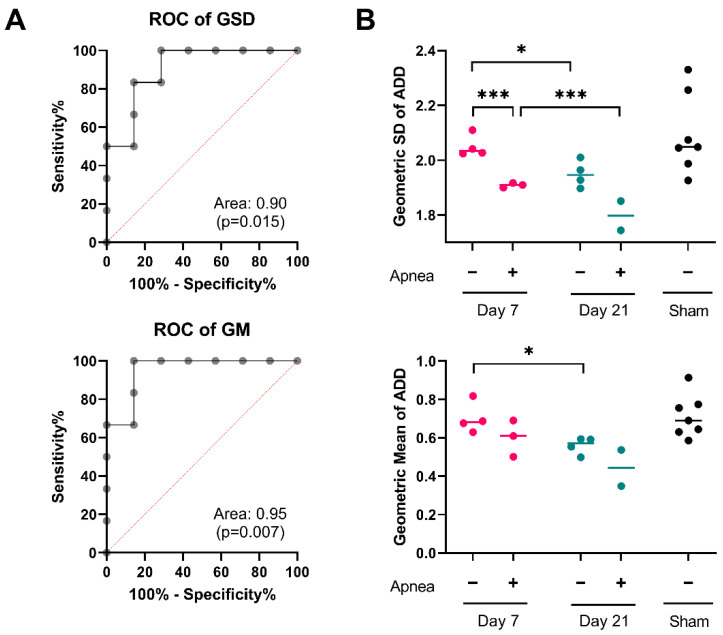
ADD signatures in individual mice. (**A**) Receiver operating characteristic curves for geometric standard deviation (GSD) and geometric mean (GM) of ADDs at 21 days post injury. (**B**) Changes in the GSD and GM of ADDs in individual mice after injury. Two-way ANOVA for the effects and interactions between post-injury apnea and survival after injury on the GSD of the ADD revealed significant contribution of both post-injury apnea, F_1,9_ = 32.86; *p* < 0.001; and post-injury interval, F_1,9_ = 13.45, *p* = 0.004. Mice that experience apnea showed evidence of a lower GSD of the ADD at day 7, t = 3.11, *p* < 0.001; but not at day 21 t = 3.11, *p* = 0.066. There was significant reduction in the GSD from day 7 to day 21 for both the apnea group, t = 2.76, *p* < 0.001, and the no apnea group, t = 3.19, *p* = 0.028. Similarly, the two-way ANOVA for the effects and interactions between post-injury apnea and survival after injury on the GM of the ADD revealed significant contribution of both post injury apnea, F_1,9_ = 5.27; *p* = 0.044; and post-injury interval, F_1,9_ = 9.10, *p* = 0.009. Mice that experience apnea did not show evidence of a different GM of the ADD at day 7, t = 1.54, *p* = 0.171 or at day 21 t = 1.75, *p* = 0.139. There was significant reduction in the GM from day 7 to day 21 for the no apnea group, t = 3.106, *p* = 0.029, but not in the apnea group, t = 1.59, *p* = 0.206. *, *p* < 0.05; ***, *p* < 0.001.

In the previous section we have demonstrated that IA-TBI is associated with specific long-term changes in several features of the ADD, including the geometric average axon diameter (GM) and their dispersion (GSD). To explore whether these ADD features can be used to discriminate injured from non-injured mice at 21 days, we performed sensitivity/specificity analyses by plotting receiver operating characteristic (ROC) curves for GM and GSD (Figure 4A). For both GM and GSD, the area-under-the curve (GM: 0.95, with CI_95%_ 0.84–1.00, *p* = 0.007; GSD: 0.91, with CI_95%_ 0.74–1.00, *p* = 0.015) indicates significant discriminatory accuracy. We also wanted to explore whether GM and GSD can be used for clinicopathological correlations, for example with the severity of injury as indicated by the presence of post-injury apnea. We subdivided injured cases based on the presence or absence of apnea, and we analyzed differences in GM and GSD values with two-way ANOVA. Despite a small number of cases, we found that both time after injury and the presence of post-injury apnea were significantly related to changes in ADD features (Figure 4B).

## 3. Discussion

White matter makes up to 30% of the human brain [10] and abnormalities of the white matter, mostly based on neuroimaging, have been implicated in a wide spectrum of neurological and psychiatric diseases. Therefore, it is important to characterize the baseline characteristics of white matter microstructure and how these are affected in different pathological conditions. The distribution of axon calibers within individual white matter tracts seems to be one such feature and here we present the first focused attempt at characterizing ADD changes in the course of traumatic axonopathy. We found that IA-TBI which leads to partial degeneration of the CST, is also associated with significant changes in ADD that occur after the resolution of the degenerative cycle early post injury. These changes include a reduction in both the average axon caliber and the dispersion of caliber values.

### 3.1. Axon Calibers and Lognormal Distribution

One of our primary observations is that axon calibers of the mouse CST have a skewed distribution with a heavy right tail, best described as lognormal. Whereas the majority of axons have small- or medium-size calibers (50% of CST axons are <0.7 μm in diameter at baseline), axons at the top 10% have calibers that are much larger than expected in a normal distribution (at baseline their calibers are >1.3 μm). This pattern is observed across white matter tracts and species, and has been associated with the need to balance two important and competing factors, the requirement for optimal information rates on one hand and the associated metabolic cost on the other [1,11]: large-caliber axons transmit greater volumes of information because information rate is linearly proportional to the axon diameter (∝ *d*) and, at the same time, they are more costly because metabolic cost is proportional to the volume of the axon (∝ *d*^2^). In other words, for a given white matter tract, the relative proportion of small- and large-caliber axons can be said to be optimized by the need to maintain functionally appropriate information rates at a minimal information cost [1]. For this reason, differences in the distribution of axon diameters, i.e., skewness and dispersion, among different white matter tracts may reflect heterogeneity of information rates conveyed by different systems [1].

The lognormal distribution of axon calibers presents an important deviation from other areas in biology where the values of a variable vary symmetrically around a mean value and have the shape of a normal (Gaussian) distribution, which is characterized by the arithmetic mean and standard deviation. Lognormal distributions, on the other hand, arise when, not the variable itself, but, its logarithm follows a normal distribution, and are best characterized instead by the GM and GSD. Whereas the variability in a normal distribution is due to independent additive effects, in the lognormal distribution variability arises mostly from independent multiplicative effects [12]. In complex biological systems such as the nervous system, occurrence of lognormal distributions can therefore be explained by the multiplicative and synergistic nature of the interactions of their elements [13].

The lognormal profile of ADD in CST is consistent with a large body of work showing that across different white matter tracts and species, axon population calibers conform to such distributions [1,14]. Similar lognormal distributions are also observed in the sizes of spines on dendrites, synaptic weights, in the firing rates of disparate neuronal populations across different environments/contexts and even in connectivity patterns between brain [13]. The ubiquity of the same type of distribution across multiple neural properties and scales of observation is not surprising, however, due to the natural interrelation between structure and function: cell body size is correlated with axon caliber which is in turn correlated with synaptic weights and firing frequency and is directly proportional to information transmission rate [1,14,15,16,17]. Therefore, the distribution of axon calibers may be driven by factors acting at multiple levels and scales of organization.

### 3.2. ADD Changes after Injury

The main finding of our study is that TBI induces significant changes in ADD early and late post injury. Although we couldn’t detect significant changes in ADD features in the first week after IA-TBI, we found that there are reductions in axons of all calibers, while large axons in the upper half of the population also exhibit some size-dependent vulnerability. It is not possible to discern whether these changes are purely due to size-dependent axon degeneration, caliber changes in surviving axons, or a combination of the two. However, the most dramatic change in ADD in our study happens between the second and third week after injury, in the absence of detectable ongoing axon degeneration. Given that the number of axons between day 7 and day 21 did not appear to change, the observed reductions in GM and GSD are more likely explained by the preferential atrophy of large-caliber axons resulting in an apparent reciprocal increase in the frequency of smaller axons. A caveat is that axonal pathology has been occasionally observed months after single injury [18,19], and conversely limited regenerative sprouting in the CNS has also been observed after TBI [20], and, therefore, we cannot rule out that at least a minor component of the observed ADD changes may relate to changes in the axonal population.. Nevertheless, a similar leftward shift in the ADD has also been reported in myelinated axons after TAI in the corpus callosum, a finding suggesting predominant loss or atrophy of large axons [21]. Similar vulnerability of larger axons and neurons is reported after ischemia [22] as well as in models of neurodegeneration [23,24].In these studies, changes in ADDs were not formally assessed, but reported changes are in keeping with our observations. While this trend indicates that similar changes in ADDs may occur in response to disparate insults and across different white matter tracts, future assessment of ADD changes with different TAI models, and across different tracts and longer survival intervals will be important in order to further validate our findings in more diverse contexts. Similarly, the ADD measure would need to be explored as a function of other parameters not assessed in our study, such as injury severity, age and sex, and it is likely that such work will yield important insights.

### 3.3. Potential Mechanisms Underlying ADD Changes

Mechanisms of ADD changes may include factors intrinsic to individual axons and extrinsic factors operating at the axon population level. The former may include bottom-up changes in protein expression, and transport or phosphorylation and turnover of neurofilaments, i.e., the main molecular determinants of axon caliber, or changes in microtubule dynamics [25,26]. On the other hand, changes in ADD may also reflect the influence of top-down factors that operate at the axon population level, such as alterations in the functional organization and connectivity in response to injury. For example, based on neural network modeling, different types of adaptive learning may be associated with distinct patterns of distributions of synaptic weights [27]. Hebbian plasticity promotes lognormal distributions, whereas homeostatic plasticity acts in the opposite direction by promoting normalization of the distribution [27].

Whether the observed ADD changes are a passive outcome of the initial injury or are associated with an adaptive mechanism related to restoration of function is beyond the remit of this study. It is of interest that the most significant ADD changes after TBI occur at later time points in the absence of ongoing degeneration, and this is also the period during which motor recovery is observed in a single-pellet reaching task (unpublished observations [28]). In contrast to diameter-based analysis of axon frequencies, rank-based analysis of axon frequencies that plots axon calibers based on order of size indicates a restoration of the original frequencies at 21 days. This pattern suggests that, if late ADD changes reflect an adaptive operation to recover an optimal state, this operation is aimed at maintaining the proportion of different ranks of axons within the population and not their absolute diameters. The biological mechanisms underlying such operations, for example changes in afferent or efferent connectivity, collateral regeneration or pruning etc. would warrant further investigation.

### 3.4. Clinical Relevance of ADD

Irrespective of mechanisms underlying the ADD changes reported here, ADD changes may also represent a signature of previous injury and serve as a measure to assess white matter pathology. Based on our sensitivity/specificity analysis, we found that ADD features are indeed able to discriminate injured from non-injured cases, and we were also able to relate ADD features with clinical parameters such as post-injury apnea. Emerging neuroimaging methods that provide estimates of axon calibers [29] such as AxCaliber3D [30,31], ActiveAx [32], oscillating gradient spin echo [33], magnetic resonance axon radius mapping [34] and others, may eventually allow the in vivo assessment of ADD changes and assess their potential as clinical biomarkers.

While these neuroimaging modalities are not capable of detecting and measuring individual axon calibers in a fashion similar to high-resolution histological methods, they offer estimates such as the “effective axon radius” [34], a compound measure of axon calibers that is heavily influenced by the long tail of the underlying ADD. Although this bias towards large-caliber axons has been considered a methodological weakness, our analysis indicates that one of the main effects of injury is the disproportional loss/atrophy of large-caliber axons, which would be preferentially detected with these MRI methods. Indeed, while we have not quantified changes in the effective radius with MR techniques, we estimate that the observed changes in the ADD after TBI would correspond to a reduction in the effective radius of by aprox. 40–50% at 21 days. Therefore, neuroimaging measures of ADD and the identification of injury-related signatures may have clinical applications in TBI and perhaps other neurological diseases in the future.

## 4. Materials and Methods

### 4.1. Experimental Subjects and Impact Acceleration TBI (IA-TBI) Model

Animals were housed in a vivarium with a 12 h light/12 h dark cycle and ad libitum access to food and water. All animal handling as well as surgical and postoperative procedures were carried out according to protocols approved by the Animal Care and Use Committee of the Johns Hopkins Medical Institutions (Protocol Number: MO19M458).

In this case, 10 to 14 week-old male C57BL/6 J wild-type mice (*n* = 9; with mean weight of 24.0 g, SD = 1.3 g) and transgenic YFP-H (B6.Cg-Tg(Thy1-YFP)HJrs/J; RRID:IMSR_JAX:003782) mice (*n* = 19; with mean weight of 24.9 g, SD = 2.8 g) were subjected to IA-TBI or sham injury as described [8,9]. Briefly, mice were anaesthetized with a mixture of isoflurane, oxygen and nitrous oxide, the cranium was exposed, a 5 mm-thick stainless-steel disc was glued onto the skull midway between bregma and lambda sutures, and a 50 g weight was dropped from 85 cm on the metal disk, while the mouse was placed on a foam mattress, with the body immobilized. This injury setting causes mild to moderate traumatic axonal injury [35,36]: there is no evident contusion at the impact site or intracranial bleeding [8], while death due to respiratory arrest is uncommon (3%). Sham animals did not receive the weight drop. Immediately after injury, the disc was removed, and the skull was inspected for skull fractures (typically <2%, *n* = 0 for this cohort). The scalp incision was closed with surgical staples. Spontaneous breathing was observed and the presence and duration of apnea or abnormal breathing was recorded. Apnea was defined to be present if it lasted more than 20 s after impact. Mean duration of apnea was 55 s (SD = 31 s). Neurological recovery was assessed by the return of the righting reflex. Mean duration of time-to-righting reflex was 234 s (SD = 123 s). No subject had apnea/irregular breathing >150 s and/or time to righting reflex >550 s, i.e., criteria that would disqualify subjects from further study to avoid hypoxic confounders. Surgical procedures and injury were performed under aseptic conditions and all animal handling and postoperative procedures were carried according to protocols approved by the Animal Care and Use Committee of the Johns Hopkins Medical Institutions.

### 4.2. Preparation of Tissues, Imaging and Morphometry

Injured and sham-injured mice were randomized to either the 7- or 21-day survival group. At each indicated survival time point, YFP-H mice were transcardially perfused with freshly depolymerized paraformaldehyde in PBS (4% in 0.1 m PBS, pH 7.4), dissected and postfixed overnight in the same fixative. Blocks containing the lower cervical spinal cord were cryoprotected (20% glycerol, 5% DMSO) and 50 μm sections at the level of C6-C7 were cut using a freezing microtome. Sections were mounted on slides, air dried and coverslipped with Vectashield ((Vector Laboratories Inc., Newark, CA, US). Sections from YFP-H mice were imaged on a Zeiss LSM 880 Confocal with Airyscan FAST Module (RRID:SCR_015963, Carl Zeiss Microscopy, LLC, White Plains, NY, US). In this case, 15 μm z-stacks covering the CST were taken at 63× objective with Airyscan FAST, deconvoluted and stitched with ZEN Black software (RRID:SCR_018163, Carl Zeiss Microscopy, LLC, White Plains, NY, US)). Images were binerised using adaptive 3D thresholding (plugin developed by Christian Henden) on FIJI (RRID:SCR_002285) [37] and individual axons were analyzed for Feret’s diameter (Supplementary Figure S2).

For electron microscopy studies, C57BL/6 J mice were transcardially perfused with 4% paraformaldehyde, 2% glutaraldehyde in 0.1 M sodium cacodylate buffer (pH = 7.2) for 30 min. Spinal cord blocks were dissected and post-fixed overnight at 4 °C in the same fixative. After rinsing in buffer for 15 min, tissues were immersed in 1% osmium tetroxide overnight. After rinsing in distilled water tissues were dehydrated in a graded ethanol series, transitioned in propylene oxide and embedded in EMbed 812 resin, using manufacturer’s recommended recipe (Electron Microscope Sciences, Hatfield, PA 14120) in BEEM capsules (reversed with cap down, Size 00). The resin was cured at 60 °C for 72 h. Semithin sections (1 μm) were cut at the level of C6-C7 and stained with 1% toluidine blue 70–90 nm thin sections were taken in the same plane as the semi-thin sections. 300 mesh Gilder Thin Bar Copper Grids (Gilder Grids Ltd., Grantham, UK, G300HS copper, EMS cat#T300-cu) were used. Grids were stained with 3% ethanolic uranyl acetate and lead citrate for 5 min and observed in a Hitachi H7600 (Hitachi High-Tech America, Inc., Schaumburg, IL, US). In each EM grid, the random superposition of the sample on the copper grid lines, allows for an unbiased sampling of the CST region. Areas of interest were identified at low magnification (4000×) at the corners and center of each hole (90 × 90 μm) in the copper grid array; and then micrographs were captured at 20,000× (10–15 images per case) and were analyzed with AxonDeepSeg for Feret’s diameter [38] (Supplementary Figure S1A).

### 4.3. Axon Diameter Distribution (ADD) Analysis

In order to assess the ADD, Feret’s diameters of axons were analyzed for each case within each experimental group by relative frequency histograms, with a bin size of 0.1μm, with Prism 9 (RRID:SCR_002798, GraphPad Software, San Diego, CA, USA). Relative frequencies per bin per case were used for fitting longnormal models for each group and for plotting average frequencies and standard error of the mean (SEM) per bin per group. For each case and each group, we estimate shape features of the ADD that may discriminate between the groups (e.g., geometric mean, geometric standard deviation) and their variance of the estimates were computed by the leave-one (mouse) Jackknife re-sampling algorithm [39]. Statistical differences between groups were assessed by ANOVA and *t*-test post hoc comparisons. *p* Values were calculated using the permutation distribution of the F-statistic and t-statistic [40], in order to retain validity with sample sizes under consideration. For estimation of axon changes with adjustment for axon losses and relative to the sham group, adjusted relative frequencies were calculated as the product of relative frequencies for each bin with the mean axon survival per group. In this case calculated standard errors account for the original variance in the relative frequencies but not for the variance in axonal survival within each group.

## Figures and Tables

**Table 1:** Changes in relative ADD features following IA-TBI.

ADD Features	Sham (*n* = 7)	Day 7 (*n* = 7)	Day 21 (*n* = 6)
Geometric mean, GM (CI_95%_)	0.69 (0.62–0.77)	0.65 (0.58–0.72)	0.51 (0.43–0.59)
Geometric standard deviation, GSD (CI_95%_)	2.10 (1.99–2.21)	2.03 (1.97–2.09)	1.97 (1.92–2.02)
Skewness (CI_95%_)	3.21 (2.79–3.62)	2.94 (2.72–3.16)	2.72 (2.55–2.88)
90th Percentile (CI_95%_)	1.79 (1.49–2.09)	1.61 (1.4–1.81)	1.22 (1.02–1.41)
Mode (CI_95%_)	0.40 (0.37–0.43)	0.39 (0.35–0.44)	0.32 (0.2–0.38)

## Data Availability

The data that support the findings of this study are available from the corresponding author, A.S.A., upon reasonable request.

## Supplementary Materials

The following supporting information can be downloaded at: https://www.mdpi.com/article/10.3390/ijms23137391/s1.
