# Supplementary material for: Long-Term Changes in Axon Calibers after Injury: Observations on the Mouse Corticospinal Tract
Source: Int J Mol Sci. 2022 Jul 2;23(13):7391. doi: 10.3390/ijms23137391 (PMC9266552; doi:10.3390/ijms23137391)
Supplement: Supplementary file 1 [file ijms-23-07391-s001.zip › ijms-1751505-supplementary.pdf]

International Journal of *Molecular Sciences*

Article

Long-Term Changes in Axon Calibers after Injury: Observations on the Mouse Corticospinal Tract

Athanasios S. Alexandris [1,*], Yiqing Wang [1], Constantine E. Frangakis [2], Youngrim Lee [1], Jiwon Ryu [1], Zahra Alam [1] and Vassilis E. Koliatsos [1,3,4,*]

[1] Department of Pathology, Johns Hopkins School of Medicine, Baltimore, MD 21205, USA; yiqingwa@andrew.cmu.edu (Y.W.); rimmie0714@gmail.com (Y.L.); jiwonr@gmail.com (J.R.); alam.zahra561@gmail.com (Z.A.)
[2] Department of Biostatistics, Johns Hopkins School of Public Health, Baltimore, MD 21205, USA; cfranga1@jhu.edu
[3] Department of Neurology, Johns Hopkins University School of Medicine, Baltimore, MD 21205, USA
[4] Department of Psychiatry and Behavioral Sciences, Johns Hopkins University School of Medicine, Baltimore, MD 21205, USA
* Correspondence: aalexa27@jhmi.edu (A.S.A.); koliat@jhmi.edu (V.E.K.)

Citation: Alexandris, A.S.; Wang, Y.; Frangakis, C.E.; Lee, Y.; Ryu, J.; Alam, Z.; Koliatsos, V.E. Long-Term Changes in Axon Calibers after Injury: Observations on the Mouse Corticospinal Tract. *Int. J. Mol. Sci.* **2022**, *23*, 7391. https://doi.org/10.3390/ijms23137391

Academic Editor: Firas Kobeissy

Received: 16 May 2022
Accepted: 29 June 2022
Published: 2 July 2022

Publisher's Note: MDPI stays neutral with regard to jurisdictional claims in published maps and institutional affiliations.

Copyright: © 2022 by the authors. Licensee MDPI, Basel, Switzerland. This article is an open access article distributed under the terms and conditions of the Creative Commons Attribution (CC BY) license (https://creativecommons.org/licenses/by/4.0/).

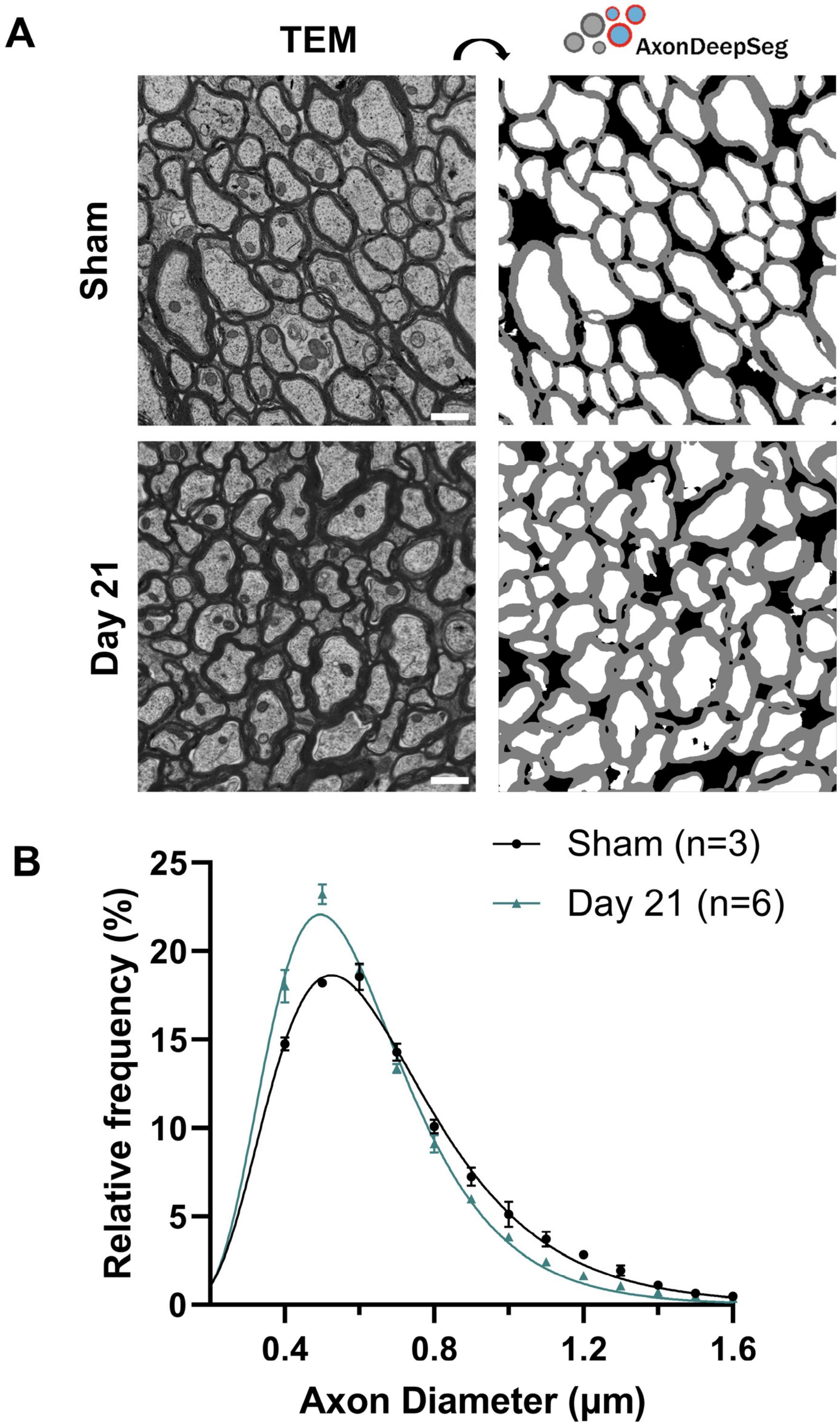

Supplementary Figure S1. Transmission electron micrographs (TEM) of corticospinal axons after sham and impact acceleration traumatic brain injury; and automated image processing for analysis of axon calibers by AxonDeepSeg. Scale bar, 600 nm. **B.** Axon diameter distributions 21 days after injury or sham-injury based on analysis of electron micrographs. Data for sham and injury groups are fitted with lognormal curves that are significantly different from each other with geometric means of 0.62 and 0.57, ($t_7 = 9.99$, $p < 0.001$), and geometric SDs of 1.50 and 1.44 ($t_7 = 4.11$, $p = 0.005$) respectively. Error bars represent standard error of the mean.

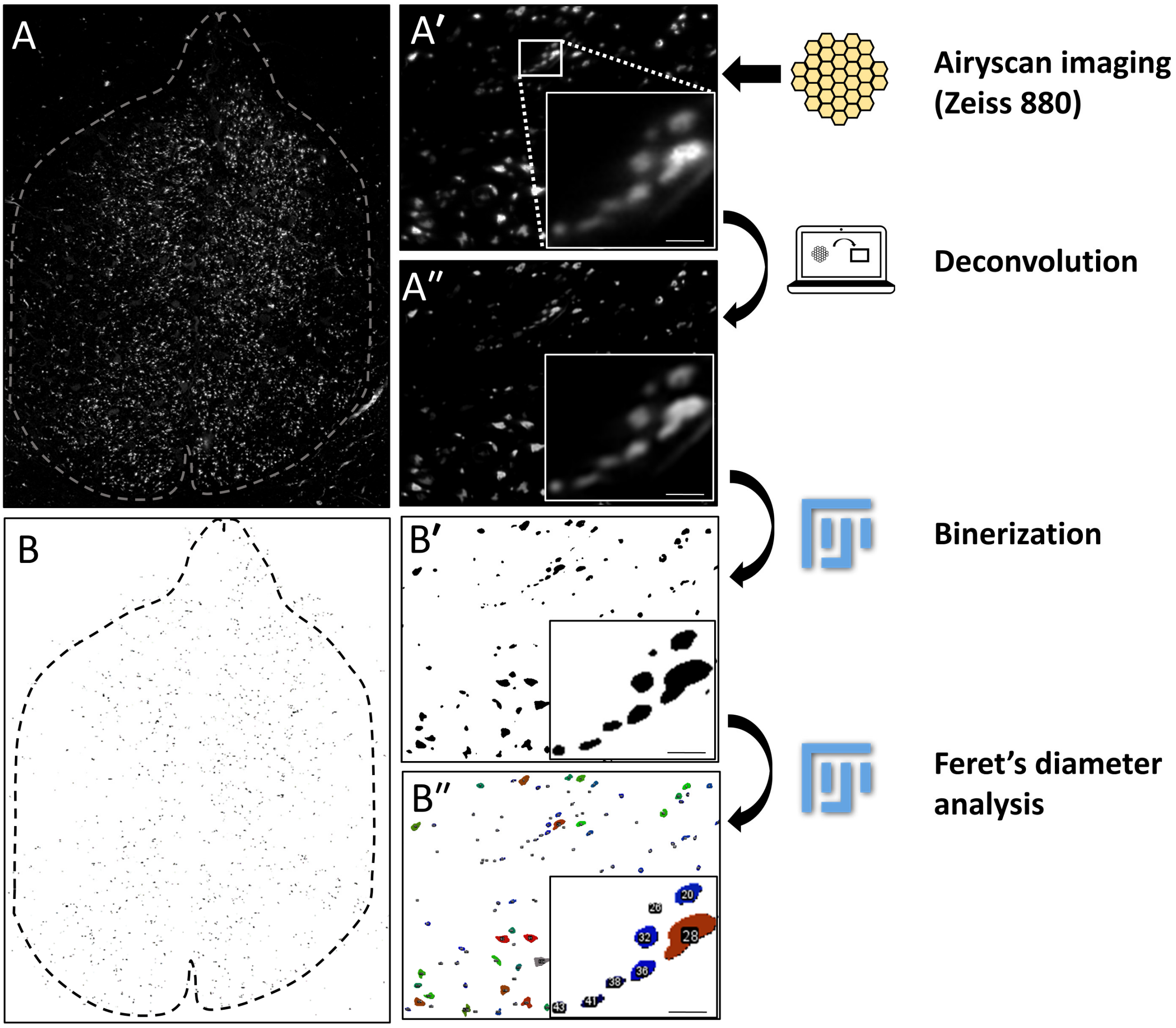

Supplementary Figure S2. Image processing for estimation of axon calibers. Tile images including the whole corticospinal tract (region indicated by dashed-line in A and B) were acquired with a Zeiss 880 Airyscan microscope (A-A′), deconvoluted (A″), and then processed in FIJI for creation of binary masks (B-B′), and particle analysis for Feret's diameter (B″; with color indicating relative diameter). Scale bar, 1 μm.
